# Early Functional Mobility After Posteromedial Release for Myelodysplastic Clubfoot in Children: Effect of Neurological Level and Quadriceps Function

**DOI:** 10.3390/children13081001

**Published:** 2026-07-29

**Authors:** Burak Abay, Alp Ozel, Ibrahim Alatas, Abdullah Eren

**Affiliations:** 1 Department of Orthopedics and Traumatology, Medical Faculty, Demiroglu Bilim University, 34381 Istanbul, Turkey; 2 Department of Physiotherapy and Rehabilitation, Faculty of Health Sciences, Bolu Abant Izzet Baysal University, 14030 Bolu, Turkey; alpozel@ibu.edu.tr; 3 Department of Neurosurgery and Spina Bifida Research Center, Demiroglu Bilim University, 34381 Istanbul, Turkey; 4 Department of Orthopedics and Traumatology, Istanbul Florence Nightingale Hospital, 34381 Istanbul, Turkey

**Keywords:** myelomeningocele, clubfoot, modified Turco posteromedial release, Functional Mobility Scale, Gross Motor Function Classification System

## Abstract

**Highlights:**

**What are the main findings?**

• Modified Turco posteromedial release was associated with improved functional mobility in children with myelodysplastic clubfoot, with gains observed across FMS distances.
• Postoperative mobility was strongly associated with neurological level and preserved quadriceps function.

**What are the implications of the main findings?**

• Functional outcomes after surgical correction should be interpreted according to the child’s underlying neurological and motor capacity, rather than deformity correction alone.
• Distance-specific functional mobility assessment may provide a more comprehensive evaluation of outcomes after surgery for myelodysplastic clubfoot.

**Abstract:**

**Background:** Myelodysplastic clubfoot is rigid, recurrent, and may limit ambulation in children with myelomeningocele. This study evaluated early functional mobility after modified Turco posteromedial release. **Methods:** Thirty-nine children (55 feet) with myelodysplastic clubfoot who underwent modified Turco posteromedial release after failed conservative treatment were retrospectively reviewed. Functional outcomes were assessed preoperatively and at final follow-up using the Gross Motor Function Classification System (GMFCS) and Functional Mobility Scale (FMS) at distances of 5, 50, and 500 m. Deformity severity, quadriceps function, complications, recurrence, and reintervention were also recorded. **Results:** Mean age at surgery was 4.2 ± 2.4 years; mean follow-up was 3.7 years. Median preoperative GMFCS was III, and median preoperative FMS total score was 7. The FMS total score improved across the Dimeglio severity groups: 10–13 (moderate), 7–10 (severe), and 4–7 (very severe). Statistically significant gains were observed at 50 m across all Dimeglio severity groups, reflecting improved school-level mobility. Children with low lumbar involvement improved across all FMS distances; those with thoracic-level involvement showed limited gains. Children with sacral involvement reached higher postoperative FMS scores. Preserved quadriceps function was associated with better functional outcomes. Recurrence occurred in 14 feet (25.4%) and early wound problems in 12 feet (21.8%). **Conclusions:** Modified Turco posteromedial release was associated with improved distance-specific mobility, particularly in children with low lumbar or sacral neurological levels and preserved quadriceps function. The GMFCS remained largely stable, with limited changes compared to FMS gains.

## 1. Introduction

Myelodysplastic clubfoot is a rigid, recurrent deformity occurring in approximately 30–50% of children with myelomeningocele [1,2,3]. Unlike idiopathic clubfoot, it is characterized by neuromuscular imbalance, sensory impairment, and abnormal motor control, which render it substantially more resistant to conservative treatment and more prone to structural recurrence [2,4]. The goal of management is not anatomical correction alone but the creation of a plantigrade, braceable, and shoeable foot that supports orthotic use, standing, transfers, and functional ambulation within the child’s existing neurological capacity [1,5].

The Ponseti method is the recommended first-line treatment for myelodysplastic clubfoot; however, recurrence rates of 33–68% have been reported in myelodysplastic feet, substantially higher than in the idiopathic form, owing to the persistent neuromuscular imbalance intrinsic to the condition [3,5,6,7]. Modified Turco posteromedial release (PMR) is an established operative option for persistent, recurrent, or rigid deformities after failed conservative management [8,9,10]. In the myelodysplastic foot, several technical adaptations are required: internal fixation is avoided owing to the risk of pin-tract infection and pressure injury in feet with impaired protective sensation, and tendons that are contracted but neurologically non-functional are excised rather than lengthened, as functional lengthening confers no biomechanical benefit in the absence of voluntary motor activity [1,8].

Despite the growing literature on deformity correction after PMR, functional mobility outcomes in myelodysplastic clubfoot remain poorly characterized. Most existing reports focus on radiographic correction and recurrence rather than on the ambulatory capacity of children in daily environments [3,5,8,9,10]. The primary aim of this study was to evaluate early distance-specific functional mobility, assessed by the Functional Mobility Scale (FMS) at 5, 50, and 500 m and the Gross Motor Function Classification System (GMFCS), following modified Turco posteromedial release in children with myelodysplastic clubfoot [11,12]. Subgroup analyses were performed to examine whether the neurological level, deformity severity, and quadriceps function were associated with the magnitude of postoperative functional gain.

## 2. Materials and Methods

### 2.1. Study Design and Patient Selection

This retrospective cohort study included children with myelomeningocele-associated clubfoot deformity who underwent surgical correction at a single tertiary referral center between January 2005 and January 2015. Institutional Review Board approval was obtained from the Clinical Research Committee of Demiroglu Bilim University prior to data collection (approval number: 10.01.2017/5611). Patients were eligible for inclusion if they had a diagnosis of myelomeningocele and clubfoot deformity, underwent operative correction of one or both clubfeet after failed conservative treatment, and had preoperative and postoperative functional mobility data. Surgical intervention was considered after failed or insufficient conservative treatment. The timing of modified Turco posteromedial release was individualized according to deformity severity, rigidity, and functional goals rather than chronological age alone. Patients with incomplete functional records or follow-up shorter than 24 months were excluded from the study.

The demographic variables included age at surgery, sex, laterality, Sharrard neurological level, and duration of follow-up. Laterality was recorded at the patient level as unilateral or bilateral. Foot-level variables included side, quadriceps function, Dimeglio severity, and surgical procedures performed during the index operation.

### 2.2. Clinical Assessment

Patients were managed within a multidisciplinary spina bifida clinic, including pediatric orthopedics, physical therapy, and neurosurgery. Surgical treatment and postoperative care were provided by a pediatric orthopedic surgeon (B.A.). Data were extracted from standardized clinical records and operative notes using a predefined data collection template. Functional scores (GMFCS and FMS) were assigned retrospectively and independently by two assessors (I.A. and A.O.). Inter-rater reliability was evaluated between the two independent assessors. Agreement was excellent for the GMFCS (weighted κ = 0.91) and good for the FMS (ICC = 0.83), supporting the consistency of the retrospective functional assessments. Formal instrumented gait analysis was not performed; functional outcomes were assessed by clinical observation and the validated scales described below. The neurological level was classified according to the Sharrard system using preoperative clinical neurological examination and manual muscle testing of the lower extremities [13,14]. Patients were categorized as thoracic, high lumbar, low lumbar, or sacral according to the lowest functional lower extremity motor level. In patients with asymmetric motor findings, the more impaired side was used for the patient-level neurological classification. Quadriceps function was evaluated using knee extension and recorded for each operated foot as absent or present. For patient-level analyses, patients were classified as quadriceps-negative if quadriceps function was absent in at least one operated foot; otherwise, they were classified as quadriceps-positive patients.

Clubfoot severity was assessed preoperatively using the Dimeglio classification, which grades deformity severity on a 20-point scale according to the reducibility of equinus, hindfoot varus, calcaneopedal derotation, and forefoot adduction, with additional points assigned for posterior crease, medial crease, cavus, and poor muscle condition [15].

According to the total Dimeglio score, the feet were classified as follows: grade I, benign or postural deformity, 0–5 points; grade II, moderate deformity, 6–10 points; grade III, severe deformity, 11–15 points; and grade IV, very severe or rigid deformity, 16–20 points. In the present cohort, all the operated feet were categorized as moderate, severe, or very severe. Dimeglio severity was recorded at the level of the operated foot. For patient-level functional analyses in bilateral cases, a higher Dimeglio grade was used to define worst-foot severity.

### 2.3. Surgical Technique

**Figure 1:**
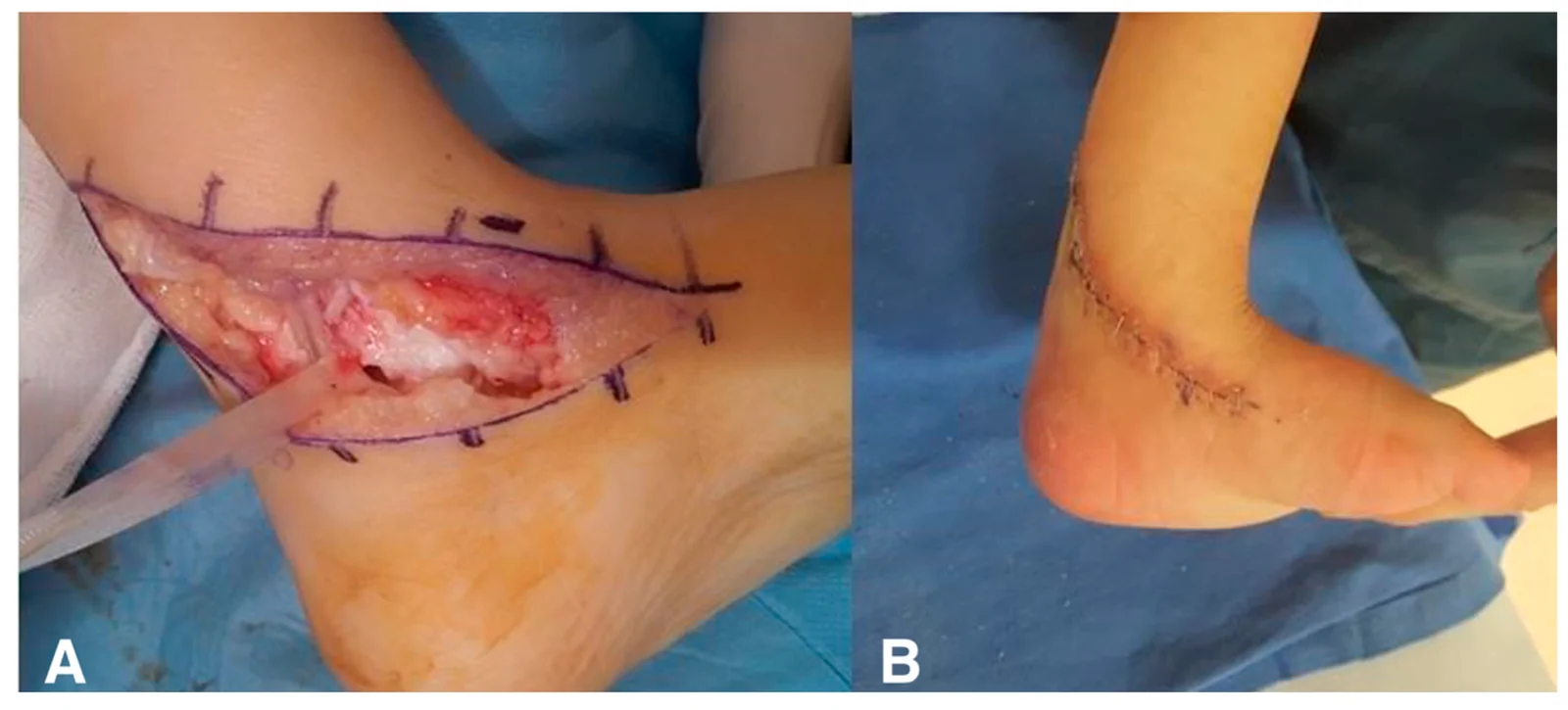
(**A**) Surgical exposure of posteromedial release performed through a modified Turco posteromedial incision for myelodisplastic clubfoot. (**B**) Early postoperative clinical appearance demonstrating successful plantigrade correction following soft-tissue balancing and wound closure.

All procedures were performed to obtain a plantigrade, braceable, and shoeable foot rather than restoring normal foot biomechanics. Surgery was performed under general anesthesia with a tourniquet. All feet underwent modified Turco posteromedial release through a single curved posteromedial (Turco) incision, extending along the medial border of the Achilles tendon, curving distally below the medial malleolus, and terminating at the plantar and medial aspect of the foot. Meticulous skin handling was maintained throughout owing to the increased risk of wound breakdown in feet with impaired or absent protective sensation secondary to myelomeningocele. Soft-tissue structures were released in a sequential posterior-to-medial order—Achilles tendon, posterior capsule, talonavicular joint, spring ligament, tibialis posterior, and plantar fascia—according to the severity and rigidity of the deformity to correct hindfoot equinus and varus, midfoot cavus, and forefoot adduction. Surgical exposure and early postoperative appearance are shown in Figure 1.

In myelodysplastic clubfoot, tendons confirmed to be neurologically nonfunctional preoperatively were excised rather than lengthened, as lengthening conferred no functional benefit in the absence of voluntary motor activity; tendons with preoperative activity were lengthened [1,8]. Intraoperative correction aimed to achieve a plantigrade, braceable foot without excessive skin tension. After the posteromedial release was completed, the necessity for additional procedures was assessed while the foot was maintained in the corrected position with an applied tourniquet. Any remaining deformity at this stage established the criteria for further intervention. Plantar fasciotomy was performed for persistent cavus or plantar soft-tissue contracture, cuboid osteotomy for fixed lateral column deformity or residual forefoot adduction, and tibialis anterior tendon transfer for dynamic imbalance or persistent supination tendency. More than one adjunctive procedure could be performed on the same foot when required.

Internal fixation was not used, and intraoperative correction was maintained using soft tissue balancing and cast immobilization alone. Routine K-wire fixation is not universally adopted in myelodysplastic clubfoot, and satisfactory outcomes have been reported following posteromedial release without routine K-wire fixation [8]. This approach was preferred because pin-tract infection and pressure injury in feet with impaired or absent protective sensation secondary to myelomeningocele make internal fixation particularly hazardous in myelomeningocele, and adequate correction was achieved through soft tissue balancing in all cases [1]. Postoperatively, the foot was immobilized in a well-padded below-knee cast in the corrected position for a total of six weeks, with a single cast change at three weeks under general anesthesia [1]. Following cast removal, custom ankle-foot orthoses were prescribed for daytime use to maintain correction and facilitate standing, transfers, and ambulation. A static AFO was routinely used as a night splint after cast removal to reduce the risk of recurrence. Postoperative rehabilitation and orthotic use were individualized according to the neurological level, quadriceps function, ambulatory potential, and skin tolerance.

### 2.4. Functional Outcome Assessment

Functional outcomes were assessed preoperatively and at the final follow-up using the Gross Motor Function Classification System (GMFCS) and Functional Mobility Scale (FMS). The GMFCS was recorded at the patient level as an ordinal measure of gross motor function. Although originally developed for children with cerebral palsy, GMFCS provides a five-level classification based on self-initiated movement, functional limitations, need for assistive devices, and wheeled mobility [12]. The Functional Mobility Scale (FMS) rates mobility at three distances—5 m (household), 50 m (school), and 500 m (community)—on a 6-point ordinal scale and has been cross-culturally adapted and validated for children and adolescents with spina bifida [11,16]. An exploratory summary measure, the FMS total score, was calculated by summing the three distance scores, which ranged from 3 to 18. This composite score has not been validated, and the primary focus remains on individual distance-specific scores.

### 2.5. Complications, Recurrence and Reoperation

Early wound problems, pressure sores, recurrence, and reinterventions were recorded at the level of the operated foot. Reinterventions included repeat modified Turco posteromedial release, talectomy, and cuboid osteotomy. The time to relapse or reintervention was recorded in months. Because two feet from the same patient may be correlated in bilateral cases, foot-level complication and reintervention rates were reported descriptively.

### 2.6. Statistical Analysis

Patient-level analyses were used for demographic variables and functional outcomes to avoid double counting bilateral cases. Foot-level analyses were used for deformity characteristics, quadriceps function, surgical procedures, complications, recurrence, and reinterventions. In bilateral cases, both feet were included in foot-level descriptive analyses; however, inferential comparisons were not performed for foot-level event rates because of the potential non-independence of feet within the same patient.

Continuous variables were summarized as mean ± standard deviation with range, median with interquartile range, or frequency with percentage, as appropriate. Ordinal functional outcomes, including GMFCS and FMS scores, were presented as median and interquartile range.

Preoperative and postoperative functional outcomes were compared using the paired Wilcoxon signed-rank test. Between-group comparisons across Dimeglio severity and Sharrard neurological level groups were performed using the Kruskal–Wallis test, and comparisons according to quadriceps function were performed using the Mann–Whitney U test. Statistical analyses were performed using R software version 4.1.2 (R Foundation for Statistical Computing, Vienna, Austria). Statistical significance was set at *p* < 0.05, and very small *p* values were reported as *p* < 0.001.

To identify independent predictors of postoperative FMS total score, a multivariable linear regression analysis was performed. The model included Sharrard neurological level, quadriceps function, age at surgery, and worst-foot Dimeglio severity as independent variables. Regression coefficients (β), 95% confidence intervals (CI), and *p* values were reported.

Given the limited sample size and the small thoracic and sacral subgroups, subgroup analyses were interpreted cautiously.

## 3. Results

A total of 39 children with myelodysplastic clubfoot deformity were included. The mean age at surgery was 4.2 ± 2.4 years (range, 2.0–12.6 years). Twenty-two patients were male (56.4%) and 17 were female (43.6%). Twenty-three patients (59.0%) had unilateral and 16 (41.0%) had bilateral involvement, yielding 55 operated feet. According to Sharrard neurological level, 4 patients (10.3%) were classified as thoracic, 14 (35.9%) as high-lumbar, 16 (41.0%) as low lumbar, and 5 (12.8%) as sacral. The median preoperative GMFCS was III (IQR, II–IV), and the median preoperative FMS total score was 7 (IQR, 5–10). The mean follow-up was 3.7 years (Table 1).

At the foot level, 29 feet (52.7%) were right-sided and 26 (47.3%) were left-sided. Quadriceps function was absent in 20 feet (36.4%) and present in 35 feet (63.6%). Dimeglio severity was moderate in 13 feet (23.6%), severe in 30 feet (54.5%), and very severe in 12 feet (21.8%). All feet underwent modified Turco posteromedial release as the index corrective procedure. Additional procedures performed during the same surgical session included plantar fasciotomy in 26 feet (47.2%), cuboid osteotomy in 8 feet (14.5%), and tibialis anterior tendon transfer in 5 feet (9.1%) (Table 2).

When patients were stratified by worst-foot Dimeglio severity, preoperative functional status differed significantly across the severity groups for GMFCS and all FMS measurements. These between-group differences persisted after surgery. The preoperative FMS total score decreased progressively with increasing Dimeglio severity, from 10 (IQR, 8–12) in the moderate group to 7 (IQR, 5–9) in the severe group and 4 (IQR, 3–6) in the very severe group (*p* < 0.001). Postoperatively, FMS total score remained significantly different across groups, with median scores of 13 (IQR, 11–15), 10 (IQR, 8–12), and 7 (IQR, 5–9), respectively (*p* = 0.009) (Table 3).

Within-group analysis showed improvement in FMS total score in all Dimeglio severity groups. The median FMS total score improved from 10 to 13 in the moderate group (*p* = 0.004), from 7 to 10 in the severe group (*p* < 0.001), and from 4 to 7 in the very severe group (*p* = 0.018). Distance-specific FMS analysis showed improvement at 50 m in all severity groups. Community-distance mobility at 500 m improved in the moderate and severe groups but not in the very severe group. In contrast, GMFCS did not significantly change within any Dimeglio severity group.

GMFCS differed significantly across Sharrard neurological levels both preoperatively and postoperatively (both *p* < 0.001) (Table 4). The median preoperative GMFCS was IV in the thoracic and high-lumbar groups, III in the low-lumbar group, and II in the sacral group. Postoperatively, GMFCS remained IV in the thoracic and high-lumbar groups and II in the sacral group, whereas the low-lumbar group improved from GMFCS III to II (*p* = 0.030). No significant within-group GMFCS change was observed in the thoracic, high-lumbar, or sacral groups. FMS scores also differed significantly across Sharrard neurological levels at all distances both preoperatively and postoperatively. The most consistent postoperative gains were observed in the low-lumbar group. In these patients, FMS 5 m improved from 4 (IQR, 3–5) to 5 (IQR, 4–6; *p* = 0.008), FMS 50 m from 4 (IQR, 3.25–5) to 5 (IQR, 3.25–5; *p* = 0.003), FMS 500 m from 1 (IQR, 1–1) to 3 (IQR, 1.5–3.75; *p* = 0.002), and FMS total score from 8.5 (IQR, 5.5–9.75) to 13 (IQR, 10.25–14; *p* = 0.001). Sacral level patients also showed measurable gains in FMS 50 m, FMS 500 m, and FMS total score, with the total score improving from 14 (IQR, 11.5–15) to 18 (IQR, 17–18; *p* = 0.042). High-lumbar patients improved in FMS 50 m and FMS total score; FMS 5 m did not reach significance (*p* = 0.074), and FMS 500 m remained unchanged. Thoracic-level patients showed no significant improvement in any FMS distance or total score.

Patients with preserved quadriceps function showed significantly better functional status than those with absent quadriceps function both preoperatively and postoperatively. Preoperatively, the quadriceps-present group had better GMFCS [III (IQR, II–III) vs IV (IQR, III–IV); *p* = 0.001] and higher FMS total score [8 (IQR, 7–13) vs. 5 (IQR, 3–7); *p* = 0.002]. Postoperatively, these differences persisted: GMFCS remained better in the quadriceps-present group [III (IQR, II–III) vs. IV (IQR, III–IV); *p* = 0.001], and FMS total score was higher [12 (IQR, 10–14) vs. 6 (IQR, 5–10); *p* < 0.001]. Distance-specific FMS scores at 5 m, 50 m, and 500 m were also significantly higher in the quadriceps-present group at both timepoints (Table 5).

Multivariable linear regression analysis identified low-lumbar and sacral neurological levels, as well as preserved quadriceps function, as independent predictors of postoperative FMS total score (Table 6). Age at surgery and worst-foot Dimeglio severity were not independently associated with the outcome. The regression model explained 68% of the variance in postoperative FMS total score (adjusted R^2^ = 0.59; overall model *p* < 0.001).

**Figure 2:**
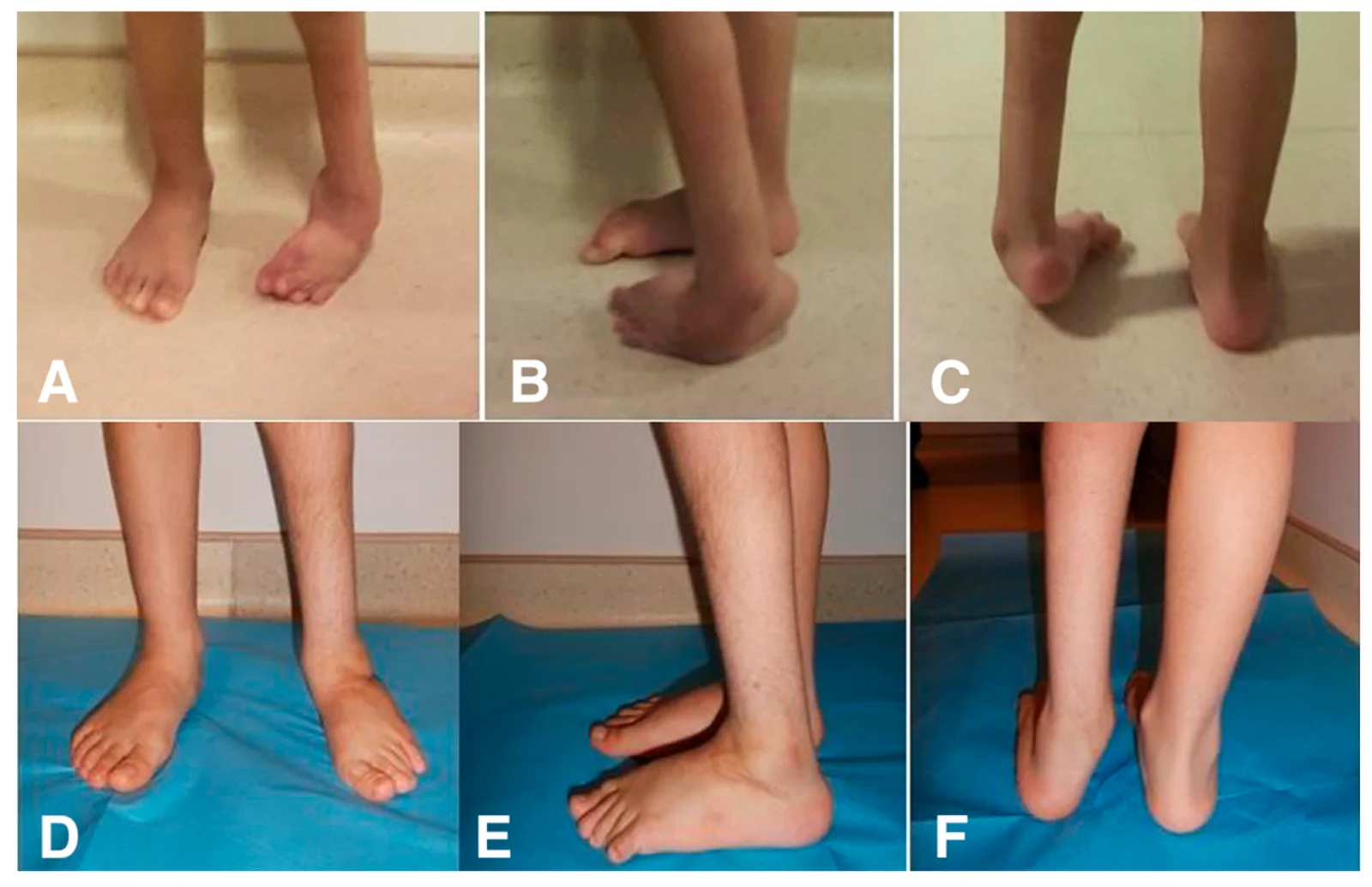
Preoperative and postoperative clinical appearance of myelodysplastic clubfoot after single-stage modified Turco posteromedial release in a 5-year-old child. (**A**–**C**) Preoperative clinical images demonstrating severe equinovarus deformity with hindfoot varus and forefoot adduction. (**D**–**F**) Clinical appearance 20 months postoperatively showing restoration of a plantigrade foot and improved hindfoot alignment.

Foot-level complications and reinterventions were recorded. Early wound problems occurred in 12 feet (21.8%), including pressure sores in 6 feet (10.9%). Recurrence was observed in 14 feet (25.4%) during follow-up. Repeat modified Turco posteromedial release was performed in eight feet (14.5%), talectomy in three feet (5.5%), and cuboid osteotomy in two feet (3.6%). The median time to relapse or reintervention was 11 months (IQR, 6–27). A representative preoperative and postoperative clinical example is shown in Figure 2.

## 4. Discussion

The main finding of this study was that modified Turco posteromedial release for myelodysplastic clubfoot was associated with measurable early improvement in distance-specific functional mobility, whereas the GMFCS level remained relatively stable at the final follow-up. This pattern suggests that surgery improved the ability to use existing motor potential after obtaining a plantigrade, braceable foot without substantially changing the global gross motor classification. PMR was associated with improved FMS scores across all Dimeglio severity groups, supporting its role as a surgical option when the goal is to obtain a corrected foot that facilitates functional mobility rather than the anatomical correction of the foot. Recurrence after Ponseti treatment in myelodysplastic clubfoot is substantially more common than in idiopathic feet; a recent systematic review reported a pooled recurrence rate of approximately 62%, and PMR may be presented to families as a treatment that may improve early braceability while still requiring long-term follow-up [3,5,8,9,10].

Neurological level appeared to be strongly associated with postoperative functional outcomes. Children with low lumbar involvement improved across all FMS distances, sacral-level patients achieved near-maximal postoperative scores, high lumbar patients demonstrated selective gains at school distance (50 m) and total score, whereas thoracic-level patients showed no significant functional gain. This supports the concept that PMR may improve the functional use of a corrected foot only when sufficient ambulatory potential exists. Deformity severity influenced baseline function but did not preclude functional improvement. FMS total score improved in moderate, severe, and very severe Dimeglio groups. Even rigid myelodysplastic deformities may benefit from surgical correction when a plantigrade, braceable foot is the goal, consistent with the outcomes reported in neuromuscular and myelodysplastic clubfoot series [8,9,10]. Therefore, postoperative outcomes should be interpreted within the child’s neurological level and motor capacity rather than according to the Dimeglio grade. This observation was supported by multivariable regression analysis, which identified low-lumbar and sacral neurological levels, together with preserved quadriceps function, as independent predictors of postoperative functional mobility. In contrast, age at surgery and deformity severity were not independently associated with postoperative FMS total score. These findings further emphasize that neurological status is a more important determinant of postoperative functional mobility than deformity severity. This interpretation is also consistent with the established influence of neurological level and muscle function on ambulatory potential in children with myelomeningocele [13,17,18].

Preserved quadriceps function was associated with better functional outcomes at all FMS distances. Children with active quadriceps exhibited higher FMS scores both before and after surgery, consistent with the known role of knee extension in walking stability and brace use [13,17,18]. These findings are consistent with the myelomeningocele functional classification (MMfC) proposed by Dias et al., which uses quadriceps function as a determinant of ambulatory capacity and may offer a more disease-specific framework for interpreting surgical outcomes [13].

Despite measurable functional gains, recurrence and wound complications remained common. Recurrence occurred in 14 feet (25.4%) and wound or pressure-related problems in 12 feet (21.8%). These rates reflect persistent muscle imbalance, sensory loss, and biological recurrence of myelodysplastic clubfoot. Careful skin closure, well-padded casting, and wound inspection at the scheduled cast change are important, given the impaired sensation in these children [1,17,19]. Early recurrence predicts further relapse; therefore, long-term follow-up is essential [8,9]. Postoperative management within a multidisciplinary framework should address deformity correction, skin protection, brace tolerance, and early treatment of recurrence. The recurrence rate (25.4%) was lower than that reported after Ponseti treatment and comparable to previous posteromedial release series, although differences in study design and follow-up preclude direct comparison [3,8,9].

May et al. reported reoperation in 41% of spina bifida feet at a mean follow-up of 4.6 years, with 24% ultimately requiring talectomy [9], suggesting that the recurrence and reintervention rates observed in the present series likely underestimate the true long-term burden given the shorter follow-up. Published PMR series consistently report higher recurrence in myelodysplastic than in idiopathic clubfoot, driven by the persistent neuromuscular imbalance intrinsic to the condition [8,9]. GMFCS captures global gross motor function, which is largely determined by neurological level and resistant to change after focal surgery; FMS reflects distance-specific mobility in real-world settings and is more sensitive to the incremental gains a corrected, braceable foot enables [11,12,16]. Flynn et al. found no significant effect of neurosegmental level on outcome using de Carvalho Neto anatomical criteria [8], a divergence that likely reflects the insensitivity of anatomical scales to functional differences that distance-specific instruments can detect. Plantar fasciotomy is frequently required when posteromedial release alone fails to address residual plantar contracture in severe and very severe deformities [1,8]; because surgery, adjunctive procedures, bracing, and rehabilitation are routinely combined, functional gains in retrospective series reflect the combined treatment approach rather than any single component.

### Limitations

This study has several strengths. It focused on functional mobility rather than anatomical correction alone and used both GMFCS and distance-specific FMS scores to evaluate household, school, and community mobility. Clinically relevant factors, including neurological level, deformity severity, and quadriceps function, were analyzed, and patient-level functional analyses were used to avoid double-counting bilateral cases.

This study also has limitations. Concomitant lower-limb deformities, such as hip dislocation or knee contracture, were not systematically controlled and may have affected FMS scores. The 10-year accrual period may have introduced temporal heterogeneity related to changes in clinical practice over time, although all patients were treated at a single tertiary center using a consistent surgical technique. Radiographic, gait, and patient-reported outcomes were not included. Because surgery was performed at a mean age of 4.2 years with a mean follow-up of 3.7 years, some mobility gains may reflect motor maturation rather than surgery alone, and the absence of a non-operative comparison group limits causal attribution. Adjunctive procedures, bracing, and rehabilitation were frequently combined with PMR; therefore, the functional gains reflect the overall treatment package rather than soft-tissue release alone. FMS scores were assigned retrospectively from records, which may introduce information bias, although independent dual scoring was used to reduce this risk. The FMS total score was interpreted descriptively only. Late recurrence and delayed skin complications may be underestimated given the early-to-midterm follow-up. Some subgroup analyses were based on small sample sizes and should be interpreted cautiously. Future prospective studies with standardized functional, radiographic, gait, and patient-reported outcomes are needed.

## 5. Conclusions

Modified Turco posteromedial release for myelodysplastic clubfoot was associated with improved distance-specific functional mobility, particularly in children with low lumbar or sacral neurological levels and preserved quadriceps function. These findings suggest that correction surgery may improve the use of existing motor potential through creation of a stable, plantigrade, and braceable foot after failed conservative treatment. Long-term follow-up within a multidisciplinary framework remains essential to maintain correction, protect skin integrity, and monitor for recurrence. These findings provide preliminary clinical evidence supporting the potential functional benefits of modified Turco posteromedial release; however, confirmation in larger prospective multicenter studies is warranted.

## Figures and Tables

**Table 1:** Patient-level baseline characteristics and preoperative functional status.

Variable	Overall (*n* = 39)
Age at surgery, years, mean ± SD (range)	4.2 ± 2.4 (2–12.6)
Sex, *n* (%)	
Male	22 (56.4)
Female	17 (43.6)
Laterality, *n* (%)	
Unilateral	23 (59.0)
Bilateral	16 (41.0)
Sharrard neurological level	
Thoracic, *n* (%)	4 (10.3)
High lumbar, *n* (%)	14 (35.9)
Low lumbar, *n* (%)	16 (41.0)
Sacral, *n* (%)	5 (12.8)
Preoperative functional status	
Preoperative GMFCS, median (IQR)	III (II–IV)
Preoperative FMS 5 m, median (IQR)	3 [2–5]
Preoperative FMS 50 m, median (IQR)	3 [2–4]
Preoperative FMS 500 m, median (IQR)	1 [1–2]
Preoperative FMS total [3–18], median (IQR)	7 [5–10]
Follow-up duration, years, mean (range)	3.7 (2.0–10.1)

Notes: Values are presented as mean (range), median (IQR), or *n* (%), as appropriate. GMFCS and FMS were recorded at the patient level. FMS scores at 5, 50, and 500 m reflect household, school, and community mobility, respectively; the total score ranges from 3 to 18.

**Table 2:** Foot-level deformity characteristics and index surgical procedures.

Variable	Overall (*n* = 55)
Foot-level baseline characteristics	
Side, right, *n* (%)	29 (52.7)
Side, left, *n* (%)	26 (47.3)
Quadriceps function, absent (–), *n* (%)	20 (36.4)
Quadriceps function, present (+), *n* (%)	35 (63.6)
Dimeglio severity	
Moderate, *n* (%)	13 (23.6)
Severe, *n* (%)	30 (54.5)
Very severe, *n* (%)	12 (21.8)
Index surgical procedures, *n* (%) *	
Posteromedial release	55 [100]
Plantar fasciotomy, *n* (%)	26 (47.2)
Cuboid osteotomy, *n* (%)	8 (14.5)
Tibialis anterior tendon transfer, *n* (%)	5 (9.1)

Notes: Values are presented as *n* (%). The unit of analysis is the operated foot (*n* = 55). In bilateral cases, two feet from the same patient were included in foot-level descriptive analyses and may not be statistically independent. Quadriceps function and Dimeglio severity were recorded at the operated-foot level. * More than one procedure could be performed on the same foot during the index surgery.

**Table 3:** Patient-level functional outcomes stratified by worst-foot Dimeglio severity (*n* = 39).

Outcome	Time	Moderate (*n* = 10)	Severe (*n* = 20)	Very Severe (*n* = 9)	*p* (Between Groups) †
GMFCS	Preop	III (II–III)	III (III–IV)	IV (III–IV)	<0.001
	Postop	III (II–III)	III (II–IV)	IV (III–IV)	0.004
	*p* (pre vs. post) *	0.317	0.083	0.248	
FMS 5 m	Preop	4 [3–5]	3 [2–4]	2 [1–3]	<0.001
	Postop	4 [3–6]	4 [3–5]	3 [2–4]	0.006
	*p* (pre vs. post) *	0.157	0.012	0.031	
FMS 50 m	Preop	4 [3–5]	3 [2–4]	1 [1–2]	<0.001
	Postop	5 [4–6]	4 [3–5]	3 [2–4]	0.002
	*p* (pre vs. post) *	0.008	<0.001	0.002	
FMS 500 m	Preop	2 [1–3]	1 [1–2]	1 [1–1]	<0.001
	Postop	3 [2–4]	2 [1–3]	1 [1–2]	0.041
	*p* (pre vs. post) *	0.012	<0.001	0.109	
FMS total [3–18]	Preop	10 [8–12]	7 [5–9]	4 [3–6]	<0.001
	Postop	13 [11–15]	10 [8–12]	7 [5–9]	0.009
	*p* (pre vs. post) *	0.004	<0.001	0.018	

Notes: Values are presented as median (IQR). Worst-foot Dimeglio severity was used for patient-level grouping to avoid double counting of bilateral cases. * Within-group preoperative-to-postoperative comparisons were performed using the paired Wilcoxon signed-rank test. † Between-group comparisons at each timepoint were performed using the Kruskal–Wallis test. Very small *p*-values are reported as *p* < 0.001. GMFCS, Gross Motor Function Classification System; FMS, Functional Mobility Scale.

**Table 4:** Patient-level GMFCS outcomes according to Sharrard neurological level (*n* = 39).

Outcome	Time	Thoracic (*n* = 4)	High Lumbar (*n* = 14)	Low Lumbar (*n* = 16)	Sacral (*n* = 5)	*p* †
GMFCS	Preop	IV (IV–V)	IV (III–IV)	III (II–III)	II (I–II)	<0.001
	Postop	IV (IV–V)	IV (II–IV)	II (II–III)	II (I–II)	<0.001
	*p* *	1.000	0.157	0.030	0.317	
FMS 5 m	Preop	1 (1–2)	1 (1–2)	4 (3–5)	5 (4–6)	<0.001
	Postop	2 (1–3)	2 (2–4)	5 (4–6)	6 (5–6)	<0.001
	*p*	0.157	0.074	0.008	0.157	-
FMS 50 m	Preop	1 (1–1)	2 (1–4)	4 (3.25–5)	6 (6–6)	<0.001
	Postop	1 (1–1)	2 (2–3)	5 (3.25–5)	6 (6–6)	<0.001
	*p* *	1.000	0.003	0.003	0.034	—
FMS 500 m	Preop	1 (1–1)	1 (1–1)	1 (1–1)	4 (2–4.5)	<0.001
	Postop	1 (1–1)	1 (1–1)	3 (1.5–3.75)	6 (5–6)	<0.001
	*p* *	1.000	0.157	0.002	0.041	—
FMS total (3–18)	Preop	3 (3–3)	5 (3–5)	8.5 (5.5–9.75)	14 (11.5–15)	<0.001
	Postop	3 (3–3)	5 (5–8)	13 (10.25–14)	18 (17–18)	<0.001
	*p* *	1.000	0.002	0.001	0.042	—

Notes: Values are presented as median (IQR). Sharrard neurological level was analyzed at the patient level. * Within-group preoperative-to-postoperative comparisons were performed using the paired Wilcoxon signed-rank test. † Between-group comparisons at each timepoint were performed using the Kruskal–Wallis test. Very small *p* values are reported as *p* < 0.001. GMFCS, Gross Motor Function Classification System; FMS, Functional Mobility Scale.

**Table 5:** Patient-level functional outcomes according to quadriceps function (*n* = 39).

Outcome	Time	Quadriceps Absent (*n* = 18)	Quadriceps Present (*n* = 21)	*p* Value †
GMFCS	Preop	IV (III–IV)	III (II–III)	0.001
	Postop	IV (III–IV)	III (II–III)	0.001
FMS 5 m	Preop	2 (1–3)	4 (3–5)	<0.001
	Postop	3 (2–4)	5 (4–5)	<0.001
FMS 50 m	Preop	2 (1–3)	4 (3–5)	<0.001
	Postop	2 (2–4)	5 (4–5)	<0.001
FMS 500 m	Preop	1 (1–1)	2 (1–3)	0.004
	Postop	1 (1–2)	3 (2–4)	0.002
FMS total (3–18)	Preop	5 (3–7)	8 (7–13)	0.002
	Postop	6 (5–10)	12 (10–14)	<0.001

Notes: Values are presented as median (IQR). † Between-group comparisons at each timepoint were performed using the Mann–Whitney U test. Very small *p* values are reported as *p* < 0.001. Patients were classified as quadriceps-negative if quadriceps function was absent in at least one operated foot; otherwise, they were classified as quadriceps-positive. FMS scores at 5, 50, and 500 m reflect household, school, and community mobility, respectively; the total score ranges from 3 to 18. GMFCS, Gross Motor Function Classification System; FMS, Functional Mobility Scale.

**Table 6:** Multivariate predictors of postoperative Functional Mobility Scale total score (*n* = 39).

Variable	β Coefficient	95% CI	*p*-Value
**Sharrard** **neurological level**			
Thoracic (reference)	—	*—*	*—*
High lumbar	1.4	−0.8 to 3.6	0.205
Low lumbar	4.0	1.8 to 6.2	<0.001
Sacral	6.3	3.5 to 9.1	<0.001
**Quadriceps function**			
Absent (reference)	—	*—*	*—*
Present	3.1	1.4 to 4.8	0.001
**Age at surgery (years)**	−0.2	−1.1 to 2.5	0.151
**Worst-foot Dimeglio severity**			
Moderate (reference)	*—*	*—*	*—*
Severe	−1.7	−3.5 to 2.9	0.435
Very severe	−3.3	−4.4 to 0.8	0.112
R^2^ = 0.68; Adjusted R^2^ = 0.59; Model *p* < 0.001			

Notes: Dependent variable: postoperative FMS total score (range 3–18). β coefficients represent adjusted mean differences relative to the reference category. Reference categories were thoracic neurological level, absent quadriceps function, and moderate Dimeglio severity. Model R^2^ = 0.68; adjusted R^2^ = 0.59; overall model *p* < 0.001.

## Data Availability

The data presented in this study are available on request from the corresponding author. The data are not publicly available due to ethical and privacy restrictions related to patient confidentiality and institutional approval requirements.

## Abbreviations

The following abbreviations are used in this manuscript:

GMFCS	Gross Motor Function Classification System
FMS	Functional Mobility Scale
PMR	Posteromedial Release
AFO	Ankle-Foot Orthosis
IQR	Interquartile Range
MMfC	Myelomeningocele Functional Classification
